# Editorial: Autoimmune pre-disease

**DOI:** 10.3389/fimmu.2023.1159396

**Published:** 2023-02-14

**Authors:** Jennifer E. Hundt, Markus H. Hoffmann, Kyle T. Amber, Ralf J. Ludwig

**Affiliations:** ^1^ Lübeck Institute of Experimental Dermatology, University of Lübeck, Lübeck, Germany; ^2^ Department of Dermatology, University Medical Center Schleswig-Holstein, Lübeck, Germany; ^3^ Division of Dermatology, Rush University Medical Center, Chicago, IL, United States; ^4^ Department of Internal Medicine, Rush University Medical Center, Chicago, IL, United States

**Keywords:** autoimmunity, pre-disease, arthritis, skin, pemphigoid, lupus, complement, proteome

## 1 Introduction

According to the revised Witebsky postulates, diseases are of an autoimmune nature if, (i) the clinical phenotype can be reproduced through the transfer of autoantibodies and/or lymphocytes; (ii) the disease can be reproduced in experimental animal models; (iii) autoreactive T cells or autoantibodies are identified; and/or (iv) distinctive clinical observations, such as an HLA association, are found (1). These postulates mostly still hold true despite they were made 3 decades ago. However, in psoriasis, lichen sclerosus and lichen planus, which are considered chronic, non-communicable inflammatory diseases, autoreactive T cells and/or autoantibodies that potentially contribute to disease pathogenesis are also detected (2–5). Thus, rather than a clear distinction between autoimmune and chronic non-communicable inflammatory diseases, categorization of a given distinct disease may be better placed in the continuum between the two. Hence, within this research topic, we cover both “classic” autoimmune diseases, such as systemic lupus erythematosus (SLE), as well as non-communicable, inflammatory diseases with detectable autoimmunity, e.g., psoriasis.

**Figure 1:**
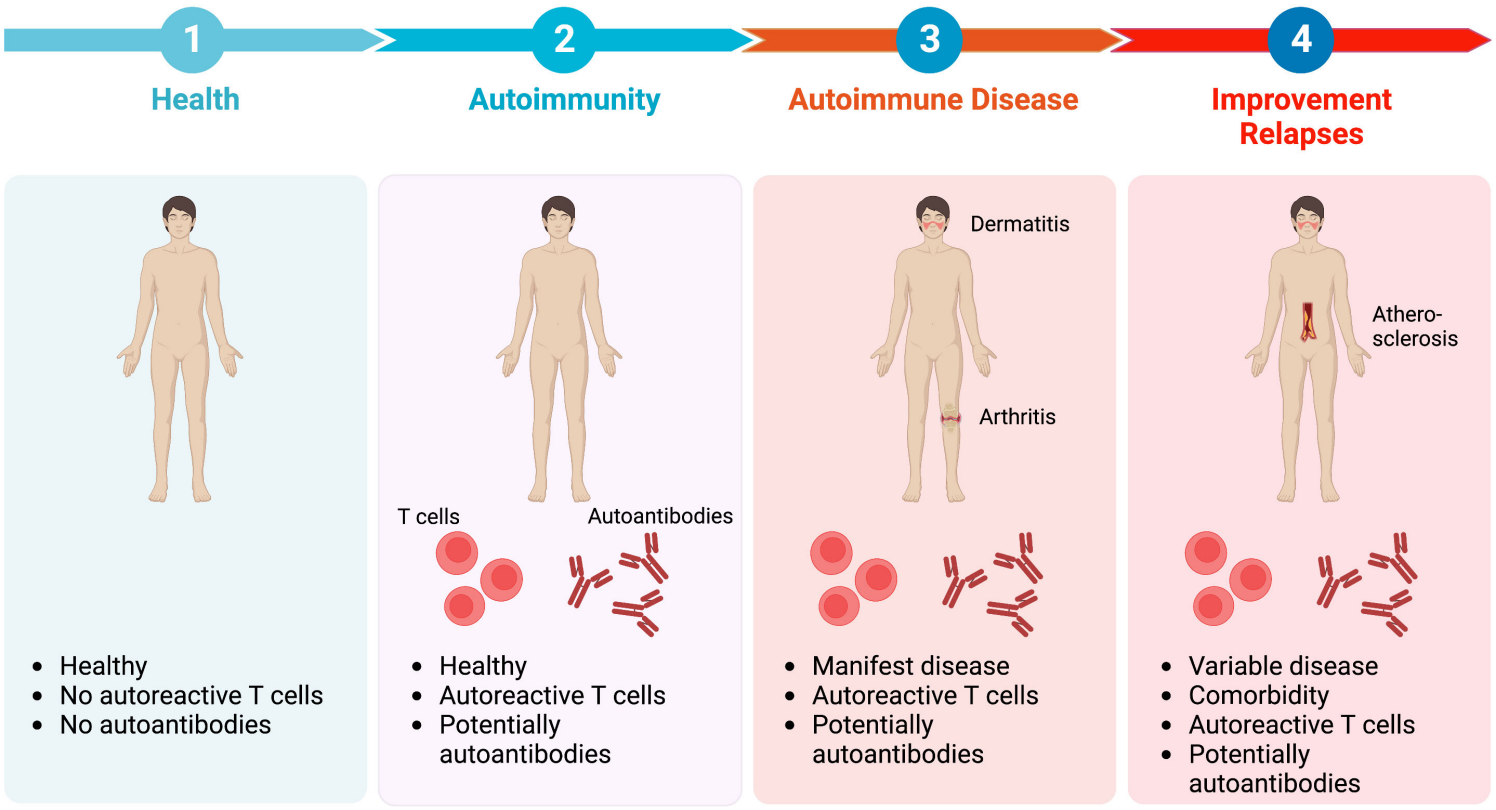
Transitional stages in the pathogenesis of autoimmune diseases. Regarding the transitional stages in the pathogenesis of autoimmune diseases, health (1) is defined by no inflammatory symptoms and absence of autoreactive T cells and autoantibodies. However, in approximately 10% of healthy individuals autoantibodies can be detected. At this stage of autoimmunity (2) no clinical symptoms are apparent. Progression towards overt autoimmune disease (3) occurs in some, but not all individuals with autoimmunity. Autoimmune disease is characterised by clinical symptoms, such as dermatitis or arthritis, as well as the detection of autoreactive T cells and/or autoantibodies. Current treatment options improve disease. However, adverse drug events as well as comorbidity are encountered frequently (4). Besides, clinical relapses of disease (flares) often occur, especially when treatment is tapered or withdrawn, because the underlying inflammatory sensitisation of tissue is sustained even during clinically silent phases (11). Image created with Biorender (www.biorender.com).

Autoimmune diseases develop over a long time, which at least spans over several years. This is maybe best demonstrated by the presence of disease-specific autoantibodies years prior to diagnosis of the corresponding autoimmune disease (6–10). The presence of disease-specific autoantibodies, does not, however, reliably predict if clinical manifestation of the corresponding autoimmune disease will occur in the future. Therefore, definite biomarkers that define the transition (i) from health to autoimmunity, (ii) from autoimmunity to autoimmune disease, and (iii) to chronicity or resolution of inflammation (**Figure 1**) would allow to implement measures to slow or prevent disease progression. This would have a significant impact on affected individuals, as well as the healthcare system because the incidence of autoimmune and non-communicable inflammatory diseases is rising and there are still many unmet medical needs in the care of patients with these diagnoses (12, 13).

In total, 25 articles covering the topic of autoimmune pre-disease were submitted to the research topic “*Autoimmune Pre-Disease*”. Ten of these articles were written by PhD- and MD-candidates from the DFG-funded Research Training Group “Autoimmune Pre Disease” (GRK 2633) that is dedicated to defining and modulating above-described transitional steps from heath to clinically manifest autoimmune disease. Some of these authors introduce themselves and their project with short clips on YouTube. For a structured description of all articles, we allocated each article to one of the following chapters: (i) From health to autoimmunity, (ii) From autoimmunity to autoimmune disease, (iii) Resolution of autoimmune disease, and (iv) New concepts in autoimmune disease.

## 2 From health to autoimmunity

In SLE, rheumatoid arthritis and type 1 diabetes (T1DM) the presence of disease-specific autoantibodies prior to clinical disease manifestation is well-established (6–10). This is less well characterized for autoantibodies targeting BP180 or BP230, which characterize and cause bullous pemphigoid (14). So far, large-scale studies demonstrated that these autoantibodies are also present in 0.9% to 2.2% of healthy volunteers (15, 16). Herein, Mai et al. review the significance of preclinical anti-BP180 autoantibodies. They conclude that in certain non-BP patient populations, the prevalence of BP180 autoantibodies is increased when compared to the appropriate controls. Some of these patient populations, e.g., neurological diseases or type 2 diabetes (T2DM), are associated with a higher risk to develop bullous pemphigoid. The increased prevalence of BP180 autoantibodies in T2DM may be confounded by the use of gliptins, oral antidiabetics that significantly increase the risk for subsequent manifestation of bullous pemphigoid (17, 18). This concept is challenged by the original data presented by Nätynki et al. demonstrating that the use of gliptins reduces levels of SDF-1/CXCL12 in bullous pemphigoid and T2DM, but does not increase autoantibodies against BP180 in diabetic patients. These findings are in line with a report from Japan (19). Hence, future studies are needed to clarify the impact of T2DM and gliptins on BP180 and BP230 autoantibodies. Schanzenbacher et al. outline that the complement system may contribute to the formation of autoantibodies. Complement can modulate autoimmune diseases at their initiation, as well as in the induction of tissue inflammation in the effector phase, as shown by Papara et al. Furthermore, shift work, circadian rhythm misalignment, and/or poor sleep may also lead to the formation of autoimmunity and autoimmune disease. These later aspects are covered in-depth in the review by Stenger et al..

Data from SLE demonstrated that cell death and dysregulated clearance of dead cells are key events in the induction of autoimmune diseases (20–22). In this Research Topic, Brieske et al. detail the impact of immunogenic cell death as driver of autoimmunity in granulomatosis with polyangiitis (GPA). GPA is an orphan anti-neutrophil cytoplasmic autoantibody (ANCA)-associated vasculitis that, in addition to small-vessel vasculitis, is characterized by granulomatous inflammation (23). The dysregulated cell death in GPA leads to the release of damage-associated molecular patterns (DAMP) such as high mobility group box 1 (HMGB1). These, along with cytokines, contribute to the loss of tolerance towards the proteinase 3 autoantigen in GPA, that ultimately lead to autoantibody formation.

In an elaborate study Anaparti et al. documented alterations in the CD4 T cell compartment of autoantibody-positive, first-degree relative of patients with rheumatoid arthritis (RA). For this, they performed multicolor flow cytometry for immunophenotyping of CD4 cells from autoantibody-negative and autoantibody positive first-degree relatives of RA patients, as well as from RA patients themselves. Herein, they identified a higher frequency of a TIGIT+ CD4 T cell subset in autoantibody-positive, compared to autoantibody-negative first-degree relatives. This underscores the importance of immunophenotyping in patients and risk populations to unravel molecular pathways in autoimmune pre-diseases (24). Zotti et al. followed a similar approach. Contrasting flow cytometric characterization of the lymphocyte compartment they found significantly reduced frequencies of recent thymic emigrants and naïve T cells, and significantly increased frequencies of central memory T cells, TH2 and TH17 cells in antinuclear antibody (ANA)+ as opposed to ANA- healthy individuals. Furthermore, CD4+ T cells in ANA+ individuals were metabolically more active than those obtained from ANA- individuals.

## 3 From autoimmunity to autoimmune disease

The article by Sprow et al. has attracted the most reads within our Research Topic. Here the researchers investigated the risk of exacerbations of dermatomyositis and SLE patients following COVID-19 vaccination. The risk for disease exacerbation following COVID-19 vaccination was 22% for dermatomyositis and 8.6% for SLE patients. Independently of these findings, the authors conclude that, given the risks of COVID-19 infection, vaccination should nevertheless be performed in most patients with autoimmune skin diseases. This latter argument is of particular importance because patients with bullous pemphigoid experience an increased risk for COVID-19-associated mortality (25). Similar findings and conclusions were made in Australian and Italian patient cohorts (26, 27). Like infections, drugs are also known to trigger autoimmune diseases; recent examples are checkpoint- or dipeptidyl peptidase 4- inhibitors (17). Anti-TNF treatment-induced psoriasis, termed paradoxical psoriasis, is among the more peculiar drug-induced autoimmune diseases, because the same class of drugs is used to treat psoriasis (28). Herein, Xie et al. review the incidence and risk factors for paradoxical psoriasis in individuals with inflammatory bowel disease (IBD) treated with anti-TNF. In a cohort of 24,547 IBD patients, paradoxical psoriasis was observed in 4.6% of cases. Risk factors for paradoxical psoriasis manifestation were female sex, younger age at anti-TNF treatment initiation, smoking, ileocolonic Crohn’s disease and use of adalimumab or certolizumab as compared to infliximab. IL-10 is another cytokine with (mostly) anti-inflammatory activity in autoimmune diseases (29). However, its’ role in autoimmunity may be more complex as highlighted in the article by Biswas et al.. The authors point out that in SLE, IL-10 seems to be also a main driver of the extrafollicular pathogenic autoantibody response. Hence, its’ pharmacological targeting needs to be well timed to achieve therapeutic effects.

Four articles within the research topic focused on an in-depth characterization of the transition from autoimmunity to autoimmune disease. Buhre et al. described the role of IgG subclass and Fc glycosylation shifts in the transition from pre- to inflammatory autoimmune diseases. They introduce the concept of a two-step model for the development of inflammatory autoimmune diseases, which isinitiated by a state of low- or non-inflammatory T/B cell responses that may shift towards more inflammatory T/B cell response. The shift to the inflammatory response is characterized by the IgG subclass distributions and IgG Fc glycosylation patterns. Hence, these might be used as biomarkers to detect this stepwise development towards autoimmune disease. Also in search of molecular markers for the development of clinical autoimmune disease important efforts were made by O’Neil et al., Niebuhr et al. and Wang et al.. In their study O`Neil et al. performed serum proteomics from first-degree relatives of patients with RA. Their analysis was stratified by the presence of absence of anti-citrullinated protein autoantibodies (ACPA). Overall, they identified 6 proteomic clusters. One of the clusters showed an enrichment of ACPA positive samples. Follow-up will determine if this dataset can be used to predict future disease onset. Wang et al. focused on Vogt-Koyanagi-Harada disease, an autoimmune inflammatory disease characterized by bilateral granulomatous uveitis, using publicly available expression datasets to identify hub genes that are potentially involved in pathogenesis. In total, six immune-related hub genes were identified. Dr. Niebuhr et al. contrasted T cell repertoires of CD45RO CD4 T cells obtained from bullous pemphigoid patients to those of age- and sex-matched controls. Interestingly, the diversity of TCR repertoires from peripheral CD4 T cells does not reflect the manifestation of bullous pemphigoid and is thus not suited to serve as a diagnostic marker.

## 4 Resolution of autoimmune disease

Once an autoimmune disease has manifested, current standard treatment options mostly aim to improve symptoms by immunosuppression, and (if needed) by hormone replacement; e.g., insulin. Current innovative treatments have significantly improved the outcomes. However, since causative treatment is not possible, these treatments must be long-term. Their adverse events and the underlying chronic inflammation may cause a significant comorbidity, especially metabolic and cardiovascular diseases. Early detection,individualization, and (in the future) potentially curative treatments will meet these medical needs in autoimmune diseases (12). Three articles of this Research Topic cover these aspects. In the paper by Song et al. the authors demonstrate that a change in reimbursement practices led to a much shorter time to remission in pemphigus patients because of early initiation of rituximab treatment. This highlights that not only break-through discoveries in translational research but also political implementation of these findings into the health care system are essential for optimal patient care. By contrast to rituximab that is effective not only in pemphigus (30, 31), but also in several autoimmune diseases (32), Zeng et al., in their meta-analysis show that there is too little evidence to support the use of curcumin and curcuma longa extract in the treatment of RA. In contrast. as reviewed by Su et al. the hedgehog signaling pathway has recently emerged as a potential therapeutic target in rheumatic diseases.

## 5 New concepts in autoimmune disease

As mentioned above, the initial definition of autoimmune diseases still mostly holds true (1), but a clear separation between autoimmunity and chronic inflammation is challenged by new methodology and new insights. Using advanced flow cytometry Polakova et al. describe the detection of rare autoreactive T cell subsets in patients with pemphigus vulgaris. Detection of (auto)antigen-specific CD4+ T cells specific for defined antigens was performed according their CD154 expression following *in vitro* stimulation (33).

In addition to these methodological innovations, thorough clinical investigations can lead to new concepts. This is exemplified by the review on anti-desmoglein autoantibodies in oral lichen planus by Didona and Hertl. Oral lichen planus is considered a chronic inflammatory disease (2). Meanwhile, autoantibodies targeting desmoglein 1 and/or 3 are hallmarks of pemphigus (34). In their review, Didona and Hertl summarize the evidence regarding the presence and potential clinical relevance of desmoglein autoantibodies in oral lichen planus. The review by Opelka et al. also focuses on autoantibodies. Here, they raise the point that autoantibodies targeting different epitopes of the same autoantigen, specifically type XVII collagen (COL17), lead to distinct clinical phenotypes: bullous pemphigoid or mucous membrane pemphigoid.

Along this line, careful clinical investigations by Baker et al. identified three patients with cutaneous pemphigus vulgaris without history of mucosal lesions. These observations challenge the concept that mucosal disease manifestations are obligatory for a diagnosis of pemphigus vulgaris (34). In the same line Sielski et al. challenge the desmoglein compensation hypothesis that correlates clinical presentation of pemphigus with the profile of autoantibodies directed against desmoglein 1 and/or 3 (35). Of note, the authors find that approximately half of active pemphigus vulgaris and pemphigus foliaceus patients investigated presented with a combination of lesion morphology and anti-Dsg3/1 levels that align with the desmoglein compensation hypothesis, whilst in the other half of the patients this correlation was not observed. However, before this dogma can be definitively refuted, prospective investigations from different sites are required.

In the article by Saurabh et al. genome-wide association studies, polygenic scores and the UK Biobank were screened in order to develop polygenic scores for autoimmune diseases. They, however, find that only comparably highly prevalent autoimmune diseases are covered by the UK Biobank, and at the same time assessed by both genome-wide association studies- and polygenic scores catalogs.

## Author contributions

All authors wrote, revised, and approve the submission of the manuscript.
